# Cardiac Aging in the Multi-Omics Era: High-Throughput Sequencing Insights

**DOI:** 10.3390/cells13201683

**Published:** 2024-10-11

**Authors:** Yiran Song, Brian Spurlock, Jiandong Liu, Li Qian

**Affiliations:** 1 Department of Pathology and Laboratory Medicine, University of North Carolina, Chapel Hill, NC 27599, USA; yrsong@email.unc.edu (Y.S.); brian_spurlock@med.unc.edu (B.S.); jiandong_liu@med.unc.edu (J.L.); 2 McAllister Heart Institute, University of North Carolina, Chapel Hill, NC 27599, USA; 3 Department of Biostatistics, University of North Carolina, Chapel Hill, NC 27599, USA

**Keywords:** cardiac aging, high-throughput multi-omics, oxidative stress

## Abstract

Cardiovascular diseases are a leading cause of mortality worldwide, and the risks of both developing a disease and receiving a poor prognosis increase with age. With increasing life expectancy, understanding the mechanisms underlying heart aging has become critical. Traditional techniques have supported research into finding the physiological changes and hallmarks of cardiovascular aging, including oxidative stress, disabled macroautophagy, loss of proteostasis, and epigenetic alterations, among others. The advent of high-throughput multi-omics techniques offers new perspectives on the molecular mechanisms and cellular processes in the heart, guiding the development of therapeutic targets. This review explores the contributions and characteristics of these high-throughput techniques to unraveling heart aging. We discuss how different high-throughput omics approaches, both alone and in combination, produce robust and exciting new findings and outline future directions and prospects in studying heart aging in this new era.

## 1. Introduction

Aging is a significant risk factor for cardiovascular disease (CVD) [1], which is a leading cause of morbidity and mortality worldwide [2]. A recent study of 5676 adults of varying ages found that accelerated heart aging is associated with a 250% increase in risk of heart failure [3]. As life expectancy rises globally, elucidating the mechanisms of cardiac aging has become a public health imperative [3]. A review of numerous aging studies delineated nine key hallmarks of aging—genomic instability, telomere attrition, epigenetic alterations, loss of proteostasis, dysregulated nutrient sensing, mitochondrial dysfunction, cellular senescence, stem cell exhaustion, and altered intercellular communication [4]—common across multiple organisms.

In cardiac tissue specifically, primary (upstream) hallmarks include oxidative stress, disabled macroautophagy, loss of proteostasis, and epigenetic alterations. Antagonistic and integrative (downstream) hallmarks, which encompass the body’s response to damage caused by the primary hallmarks and the consequences of dysregulation of those responses, respectively, include mitochondrial dysfunction, dysregulated metabolism, cellular senescence, altered cell communication, and inflammation [5,6,7]. Cardiac aging, even within the broader context of organismal aging, exerts a profound and distinct impact, leading to specific physiological changes [4,7], including cardiac hypertrophy, diastolic dysfunction, myocardial fibrosis, valvular fibrosis and calcification, arterial thickening and stiffness, and endothelial dysfunction [8,9,10]. Thus, understanding the molecular mechanisms of cardiac aging is crucial for developing effective interventions. 

The advent of Next-Generation Sequencing (NGS) technologies has revolutionized our ability to interrogate the molecular underpinnings of cardiac aging. NGS refers to high-throughput sequencing techniques that enable rapid sequencing of DNA and RNA, allowing for the comprehensive analysis of genomes, transcriptomes, and epigenomes. Since their introduction in the first decade of the 21st century, NGS technologies have evolved significantly, becoming more accurate, faster, and cost-effective. Key advancements include the development of sequencing platforms such as Illumina’s sequencing by synthesis, which dramatically increased throughput and reduced costs, and the emergence of single-cell sequencing technologies, which allow for the analysis of cellular heterogeneity within cardiac tissue. These technological advancements have made it possible to integrate multiple omics layers—genomics, epigenomics, transcriptomics, proteomics, and metabolomics—in cardiac aging studies [11], providing unprecedented insights into the molecular and regulatory landscape of both healthy and pathological hearts (Table 1). For instance, NGS-based transcriptomics enables the comprehensive profiling of gene expression changes associated with aging, while epigenomic studies reveal age-related modifications in DNA methylation and chromatin accessibility. Proteomics and metabolomics, enhanced by high-throughput mass spectrometry techniques, complement these findings by elucidating changes at the protein and metabolite levels.

**Figure 1:**
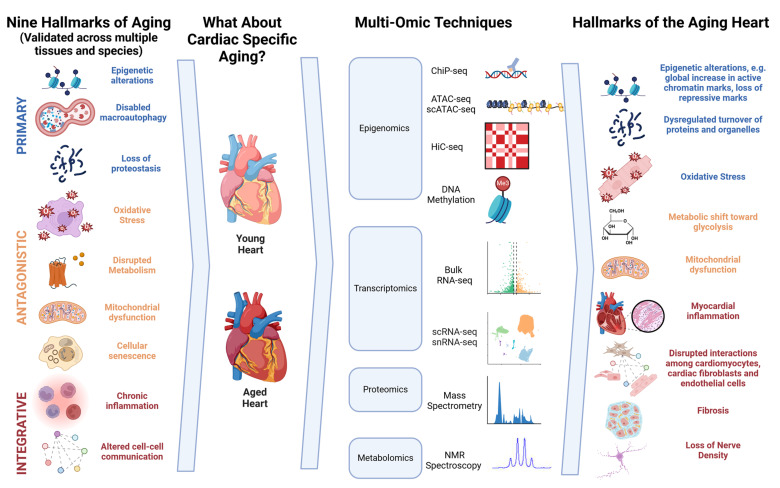
Summary figure of high-throughput sequencing in the heart aging. Primary hallmarks include upstream drivers of aging phenotypes. Antagonistic hallmarks include cellular and tissue-level responses to primary hallmarks that become dysregulated over time. Integrated hallmarks include downstream consequences of primary and antagonistic hallmarks [4].

In this review, we describe high-throughput omics techniques and then explore how they have deepened our understanding of hierarchical ordered cardiovascular aging hallmarks [6] (Figure 1). This review focuses on three key objectives: first, to analyze how each type of omics data have contributed to unraveling the molecular mechanisms and cellular processes underlying cardiac aging; second, to explore the interplay between different omics datasets and the correlations among genetic, transcriptomic, proteomic, and metabolic changes; and third, to examine the real-world applications of multi-omics approaches, such as discovering biomarkers for the early detection, progression monitoring, and prognosis of age-related cardiac conditions and identifying potential therapeutic targets. By integrating data across different omics layers, researchers can construct a holistic view of the molecular networks driving cardiac aging, leading to the identification of novel biomarkers and therapeutic targets.

## 2. Epigenomics Provides Evidence for Epigenetic Alteration in Aging Hallmarks

Epigenetic alteration is a hallmark of cardiovascular aging [4,6] and contributes to the (dys)regulation of gene expression in the aging heart independently of the DNA sequence. Altered epigenetic mechanisms of gene regulation in aging include DNA methylation, histone modification and chromatin remodeling complexes, and non-coding RNAs (ncRNAs) [38,39]. Age-associated epigenetic alterations in cardiac tissues impact the expression of genes linked to pathophysiological processes such as the loss of proteostasis, mitochondrial dysfunction, and inflammation [6]. During cardiac aging, global changes in DNA methylation patterns occur, leading to hypo- or hypermethylation of gene promoters and enhancers, which can alter gene expression profiles [39]. Histone modifications, such as acetylation and methylation, are also altered, affecting chromatin accessibility and the recruitment of transcriptional machinery [40]. Additionally, chromatin remodeling and ncRNAs, which widely regulate gene expression in multiple biological processes, also play a critical role in the epigenetic reprogramming of the aging heart [41]. Collectively, these epigenetic alterations impact cellular processes such as oxidative stress response, inflammation, and metabolic regulation, which are central to cardiac aging. Conversely, aging-associated oxidative stress can lead to alterations in chromatin structure and epigenetic markers [42]. A major consequence of oxidative stress is the loss of expression and activity of the histone deacetylase and transcriptional (co)activator Sirtuin 1 (Sirt1). Sirt1 is a major regulator of oxidative stress response pathways, creating a negative feedback loop leading to further oxidative damage and further chromatin rearrangements [43].

Epigenetic alterations impact transcriptional regulation by modifying chromatin accessibility and structure, which in turn affects the binding of transcription factors and the recruitment of the transcriptional machinery to gene promoters and enhancers [44]. In broad strokes, histone acetylation typically relaxes chromatin structure, enhancing gene expression by allowing transcription factors to access DNA, whereas histone methylation can either activate or repress transcription depending on the specific residues modified.

As noted above, epigenetic alterations are a hallmark of aging in a variety of tissues. However, cardiac aging exhibits unique patterns in the extent and specific locations of these changes. For instance, while H3K27ac increases around TSSs in both cardiac and skeletal muscle tissues, the downstream effects on gene expression differ due to tissue-specific gene regulatory networks [13]. Additionally, certain cardiac lineage genes show unique epigenetic modifications not observed in other tissues, reflecting the lineage specificity of epigenetic aging. Advancements in high-throughput sequencing technologies have enabled comprehensive studies of epigenetic alterations associated with cardiac aging to be carried out. Techniques such as Chromatin Immunoprecipitation Sequencing (ChIP-Seq), Assay for Transposase-Accessible Chromatin Sequencing (ATAC-Seq), DNA methylation analysis (e.g., bisulfite sequencing), and Hi-C sequencing provide unique insights into different aspects of the epigenome and its impact on gene expression. 

### 2.1. ChIP-Seq, Hi-C Sequencing, and DNA Methylation Analysis

ChIP-Seq maps DNA–protein interactions, specifically identifying histone modifications. By mapping these histone marks across the genome, ChIP-Seq reveals changes in chromatin accessibility and structure that influence transcriptional regulation during cardiac aging. To study DNA methylation, techniques like bisulfite sequencing and methylation arrays are commonly used. Bisulfite sequencing involves treating DNA with bisulfite to convert unmethylated cytosines to uracil, allowing for the detection of methylated cytosines during sequencing. This method provides a comprehensive view of DNA methylation patterns across the genome, particularly around transcription start sites (TSSs), which can affect gene expression by altering transcription factor binding and chromatin structure. Hi-C sequencing captures the three-dimensional organization of the genome by identifying physical interactions between chromatin regions. Alterations in chromatin conformation during aging can influence gene regulation by reorganizing chromatin domains and affecting enhancer–promoter interactions.

One study showed a significant increase in the genes lacking CpG islands (CGI− genes) in aged tissues, contributing to chronic inflammation and physiological deterioration [12]. ChIP-seq is utilized here to confirm that CGI− genes are more associated than CGI+ genes with heterochromatin, as marked by the repressive tri-methylation of histone 3 at lysine 27 (H3K27me3) [12]. This repressive mark leads to a condensed chromatin state, reducing transcriptional activity. However, during aging, changes in histone modifications can lead to the misregulation of these genes. Hi-C data suggested that CGI− genes in euchromatic domains are more susceptible to misexpression during aging due to alterations in chromatin architecture that affect transcriptional regulation. 

When combined, ChIP-Seq, RNA-Seq, DNA methylation, and metabolic analyses provide a comprehensive view of gene regulatory networks. For example, the activating acetylation of H3K27 (H3K27ac) has been shown to increase during aging [13,14]. The presence of H3K27ac at enhancers of glycolytic genes, identified by ChIP-seq, was highly correlated with RNA-seq data showing the increased expression of glycolysis-related genes (e.g., Hk2, AldoA, Ldha) in aged cardiomyocytes. Aging heart showed significant changes in the DNA methylome around TSSs, with approximately equal numbers of hypo- and hyper-methylated CpG sites, which can influence gene expression by affecting chromatin compaction and transcription factor accessibility. With data integration to identify enriched TF motifs, binding motifs for members of the zinc finger of the cerebellum (Zic) family were overrepresented in upregulated genes and genes that had an increase in H3K27ac and a decrease in H3K27me3. This indicates that histone modifications collaborate with DNA methylation changes to regulate gene expression during cardiac aging [13]. Another integration of epigenetic and metabolic (UHPLC-MS/MS) data showed that increased histone acetyltransferase activity of p300/CBP leads to enhanced H3K27ac at enhancers of glycolytic genes, increased expression of these genes, and a shift towards glycolysis in aged cardiomyocytes [14]. Indeed, chromatin accessibility in the heart broadly increases with age, primarily through gains in H3K27ac and losses in H3K27me3. 

In contrast to the heart, where aging induces comparable levels of hyper- and hypomethylation, hypermethylation predominates in other tissues like liver and quadriceps muscle [13]. Additionally, the cardiac transcriptome is more stable with age than that of the liver and changes to active and repressive marks in the heart map more neatly onto gene expression changes than those in skeletal muscle. Thus, while some epigenetic changes are common across tissues, such as increased H3K27ac and decreased H3K27me3, the impact on transcriptional regulation can be tissue-specific. 

### 2.2. ATAC-Seq and Single-Cell ATAC-Seq (scATAC-Seq)

ATAC-seq (Assay for Transposase-Accessible Chromatin using sequencing) is a NGS-based assay used to map chromatin accessibility across the genome. It utilizes the hyperactive Tn5 transposase enzyme, which inserts sequencing adapters into regions of open chromatin. By doing so, ATAC-seq identifies accessible DNA regions where regulatory proteins, such as transcription factors, can bind. This mapping of chromatin accessibility is crucial for identifying regulatory regions of the genome and understanding how gene expression is regulated epigenetically. Single-nucleus ATAC-seq (snATAC-seq) and scATAC-seq assess chromatin accessibility at the single-cell resolution, uncovering cell-type-specific epigenetic landscapes.

SnATAC-seq on adult C57BL/6 J 3-month-, 10-month- and 18-month-old male mice revealed minimal age-dependent changes in chromatin accessibility in the heart compared to various other tissues (frontal cortex, hippocampus, heart, bone marrow, and skeletal muscles) [15]. However, endothelial cells (ECs) showed tissue-dependent age-associated chromatin changes and occasional overlap with age-dependent candidate *cis-*regulatory elements (cCREs). For instance, the promoter of Nhp2 is more accessible in the ECs in the hearts of aged mice [15], suggesting increased transcriptional activity due to enhanced chromatin accessibility. This accessibility allows transcription factors to bind more readily to the Nhp2 promoter, leading to elevated gene expression, which may impact endothelial function during aging.

These findings highlight how epigenetic changes, such as modifications in histone marks and chromatin accessibility, directly influence transcriptional regulation by modulating the interaction between DNA and transcription factors. Such alterations lead to transcriptomic changes that contribute to the aging phenotype of the heart by affecting the expression of genes involved in critical cellular processes. Understanding these mechanisms provides valuable insights into the molecular underpinnings of cardiovascular aging and potential therapeutic targets.

## 3. Transcriptomics as a Link between Genomic and Proteomic Changes in Cardiac Aging

### 3.1. Bulk RNA Sequencing

Bulk RNA sequencing has been instrumental in deciphering the transcriptomic landscape of the aging heart. It measures the average gene expression across a sample, allowing the identification of differences between and among distinct conditions. This technique, although lacking the resolution of single-cell methods, offers valuable insights into the general shifts in gene expression associated with aging and is used broadly to compare how gene expression changes during aging vary by species, sex, and organ.

On the species level, cardiac aging in zebrafish initiates at mid-age (24 months), followed by a gradual progression. Similarly to other species, it is marked by increased DNA damage, inflammatory response, reduced mitochondrial function, and the accumulation of immune cells, mostly macrophages [16,17]. Indeed, zebrafish and rat hearts have shown similar aging-related expression profile changes [19], including the upregulation of circadian genes and downregulation of collagen genes [16,20]. However, the muscle-specific upregulation of autophagy-related genes and AP-1 transcription factor genes imply zebrafish-specific anti-aging characteristics [20]. Another recent study employed various ROS assays and bulk RNA-seq to investigate oxidative stress in aging rat and human hearts. They showed that increased oxidative stress during aging impacts similar pathways in both species, including those involved in mitochondrial fatty acid oxidation and sirtuin signaling [21]. 

Various age-and-sex-dependent and age–sex interaction genes have been identified, in particular genes involved in cell cycle regulation and cAMP signaling [18]. Moreover, a sex-adjusted gene set enrichment analysis of RNA seq of young and aged murine hearts revealed “unexpectedly” few enriched pathways and biological processes. Only after using the more sensitive gene set variation analysis were researchers able to link general cardiac aging to changes in exon usage in functionally coordinated genes through differential expression of RNA-binding proteins and splice factors [18]. In keeping with the sexual dimorphism of cardiac function and aging, the pregnancy hormone relaxin (Rlx) has been shown to be cardioprotective against cardiac fibrosis and atrial fibrillation. Rlx therapy on young and aged rats suppressed genes and signaling pathways associated with inflammation and heart failure (HF) in both males and females [22]. In female ventricles, relaxin also helped to reverse age-associated increases in macrophage infiltration, atrial natriuretic peptide levels, and complement cascade activation [22].

At the organ level, cardiac aging shows a number of distinctions from other tissues in comparative studies. As mentioned previously, aging cardiac ECs are epigenetically distinct from those of other organs, and the gene expression landscape is similarly unique. Murine cardiac ECs showed unique downregulated pathways related to endothelial barrier function (ECM receptor interaction, focal adhesion, and gap junction genes) and calcium signaling and upregulated pathways related to Type 1 diabetes and nicotinamide metabolism compared to ECs of the brain or kidney vascular bed. Additionally, the same study applied single-cell analyses to show that the sub-population of apelin receptor-enriched ECs attenuated with age in mouse hearts [23]. Alongside bulk RNA-seq, the ability to study gene expression in single cells has been another powerful tool in the high-throughput toolbox for studying aging in distinct cardiac cell types.

### 3.2. Single-Cell and Single-Nuclei RNA Sequencing

Single-cell and single-nuclei RNA-seq enable investigators to explore cell-type-specific and heterogeneous differences in gene expression and even recognize and compare subpopulations within certain cell types. ScRNA-seq has revealed subtle yet critical changes within cellular subpopulations during aging, which are beyond the capacity of bulk RNA analysis [23]. Cardiomyocytes, the cell type that comprises the bulk of the heart’s mass, showed a dramatic loss in cell numbers and profound transcriptional changes in aging monkeys [24]. Another major cardiac cell type, fibroblasts, showed significant changes in the expression of inflammatory, extracellular matrix organization, angiogenesis, and osteogenic genes in snRNA-seq of 18-month-old compared to 3-month-old murine hearts. Additionally, the dataset also identified an increase in a subpopulation of fibroblasts expressing osteoblast genes in the epicardial layer [25]. Aged fibroblasts impaired endothelial cell angiogenesis and autophagy and augmented the proinflammatory response by upregulating the expression of anti-angiogenic genes like Serpine1 and Serpine2 [25]. Later studies using both the same murine dataset and snRNA-seq of primate hearts identified ECs as having acquired the most senescent phenotype during aging, meaning they show the greatest upregulation of senescence marker genes [25,26,45]. At the cellular level, scRNA-seq revealed that the neurorepulsive factor semaphorin-3A (Sema3a) was repressed in hearts with senolytic treatment and that this effect occurred specifically in ECs, whereas other cell types implicated in cardiac aging, such as fibroblasts, were unaffected. At the same time, aging is associated with a decrease in microRNA 145 (miR-145) that counteracts the effects of Sema3a, contributing to endothelial Sema3a overexpression and reduced axon density, further tipping the balance toward decreased innervation. On the other hand, the removal of senescent cells by treating the mice with senolytic drugs reversed age-related losses in innervation, suggesting a potential therapeutic approach [25,26,45].

Key aging-related transcription factors (TFs) in specific cell types or sub-cell types have been identified using scRNA-seq and snRNA-seq, often paired with SCENIC (Single-Cell Regulatory Network Inference and Clustering) [46] to map transcriptional regulatory networks. SCENIC leverages co-expression analysis (via GENIE3 or GRNBoost) to identify gene sets coexpressed with transcription factors (TFs), followed by motif enrichment analysis (cisTarget) to pinpoint the TFs’ direct targets and characterize regulons. Finally, AUCell calculates the activity of these regulons in individual cells, revealing the specific roles of TFs in different cellular contexts during aging. For example, FOXO3A, a longevity-associated transcription factor, has been recognized as a master regulator gene and is downregulated in six subtypes of coronary arteries and aortic arches vascular cells during aging in monkeys [27]. Comparing published mouse heart [23] and monkey heart scRNA data [27], similar patterns, e.g., the downregulated expression of YAP1, insulin receptor, and VEGF receptor 2 and increased interaction of ECs and immunocytes, have been found in both models. Moreover, in aged cardiomyocytes, FOXP1 has been identified as a key downregulated TF associated with the dysregulation of target genes critical to heart function and cardiac diseases [24]. Finally, the TF BACH1 was identified as a master regulator of oxidative stress-related genes, contributing to cellular senescence in both mouse and monkey hearts during aging [28]. 

With the advancement of sequencing technologies and the significant reduction in costs, researchers now have access to increasingly comprehensive datasets that offer broader coverage and more frequent data collection. This shift enables the transition from simple comparisons between two or three time points to more sophisticated statistical approaches, such as regression models, where age or other variables can be treated as continuous factors influencing gene expression. For example, in human cardiac tissue from dilated cardiomyopathy (DCM) patients and healthy donor controls, a regression analysis across age and sex has revealed several aging-associated genes. In donor cardiomyocytes, TOLLIP expression was found to correlate with increasing age, while TGFBI and NFIL3 showed positive correlations with aging only in DCM cardiomyocytes, illustrating how regression models can capture subtle gene expression changes between conditions and over time [47].

As larger datasets become available, such as the comprehensive single-cell atlas of mouse tissues (Tabula Muris Senis) [48] and multi-layer transcriptome-wide profiling of porcine cardiac muscle (covering mRNA, miRNA, lncRNA, and circRNA) [49], the potential for integrating bulk RNA or scRNA data has greatly expanded. This integration allows for the application of novel statistical and machine learning models to address complex biological questions, moving beyond static comparisons to more dynamic, multifactorial analyses. Such approaches can enhance our understanding of cardiac aging by treating factors like age, disease state, and cellular composition as continuous variables within models, providing deeper insights into the molecular underpinnings of aging.

## 4. Proteomic and Metabolomic Insights into Cardiac Aging and Dysfunction

Mass spectrometry (MS)-based proteomics quantifies proteins and their modifications, such as phosphorylation and acetylation, which are crucial for understanding proteome alterations with aging. Protein arrays enable the high-throughput screening of protein interactions and functions, shedding light on the molecular mechanisms underpinning aging. In metabolomics, techniques like liquid chromatography–mass spectrometry (LC-MS) and nuclear magnetic resonance (NMR) spectroscopy provide comprehensive profiles of metabolites. These methods reveal metabolic pathways affected by aging, offering insights into metabolic dysfunctions that contribute to age-related diseases.

Utilizing UHPLC-MS, longevity-associated variant (LAV)-BPIFB4 therapy has been examined on diabetic hearts. It indicated the subtle changes in mitochondrial-related proteins and lipid metabolism that could contribute to LAV-BPIFB4-induced cardio-protection in a murine model of type-2 diabetes [29]. This research also integrates RNA-seq to investigate differentially expressed genes related to mitochondrial and metabolic function.

NMR quantified the metabolic changes. Comparing 3-month-old to 24–26-month-old mice, one study showed an increase in 4-aminobutyrate (GABA), which is involved in modulating heart rhythm and function, and a change in purine metabolites. It showed increased levels of branched-chain amino acids (BCAAs), such as leucine, isoleucine, and valine [30]. Another study compared female and male mice aged 3, 6, 12, and 24 months, further illustrating that acetic acid and BCAAs like valine and isoleucine showed a biphasic pattern, peaking at 6 months and decreasing thereafter. It also highlighted the sex differences in metabolic alterations, e.g., a male-specific increase in adenosine diphosphate (ADP) and loss of nicotinamide adenine dinucleotide (NAD), suggestive of impaired energy production, and a female-specific loss of acetylcarnitine and methionine with age [31]. Fatty acid oxidation (FAO) was shown to increase glucose metabolism in aged hearts, reflecting reduced metabolic flexibility [30]. Another study assessed the mitoxantrone (MTX) treatment effect in elderly mice. It revealed increased FAO and the oxidation of amino acids and decreased levels of glycerol and UPP-mediated proteolysis, implying metabolic adaptation to FAO and protein degradation as energy sources. These metabolic alterations may contribute to chronic cardiotoxicity and structural heart remodeling two months after MTX exposure [32]. 

## 5. Multi-Omics Approaches for a Holistic Understanding of Cardiac Aging

Multi-omics is an integrative approach that combines data from various omics disciplines to provide a comprehensive view of the biological processes and systems. It provides more solid and comprehensive understanding of the cardiac aging, by serving as a cross-validation or guidance tool.

### 5.1. scRNA-Seq and scATAC-Seq Together Help to Unravel Regulatory Networks

scRNA/snRNA and scATAC have proven to be highly complementary. Researchers use these methods for the cross-validation of transcription factor (TF) regulatory networks and open chromatin states. For instance, cardiac non-myocytes have been characterized at the single-cell transcriptome and epigenome levels in three-month-old mice, revealing distinct cellular subpopulations and identifying specific gene expression and chromatin accessibility patterns that contribute to cardiac function [50]. Additionally, studies on the aorta of mice at different ages (4, 26, and 86 weeks) have identified endothelial subpopulations that respond to cellular senescence [33]. Focusing on heart tissue from healthy aged donors, a recent study exploiting single-nuclei transcriptomics and chromatin accessibility profiling revealed changes in TF motifs, such as increased IRF1 and IRF7 motifs in aged cells [34]. This integrative approach helps in finding the regulatory networks driving cardiac aging by correlating gene expression profiles with chromatin accessibility patterns.

### 5.2. Integrating RNA-Seq and Proteomics to Unveil the Real Transcript and Protein-Level Change

RNA sequencing and proteomics are often combined as a transcript-level change does not always predict a change at the protein level in cardiac aging. A recent study used RNA-seq to identify increased exocytosis and cellular transport pathways, and decreased protein folding pathways. In addition, proteome (mass spectrometry) data revealed additional changes, such as reduced fatty acid oxidation and increased autophagy. Protein complexes showed the loss of normal stoichiometry, especially in protein homeostasis. The authors also identified genetic loci that influence age-related changes in protein homeostasis, suggesting that genetic variation can modify the molecular aging process through quantitative trait loci (QTL) [35]. 

For instance, the lncRNA Sarrah has been shown to be anti-apoptotic in cardiomyocytes. In young mice, the overexpression of Sarrah improves cardiomyocyte survival, endothelial cell proliferation, and functional recovery after myocardial ischemia. An RNA-seq analysis revealed that Sarrah is downregulated during vascular aging. To understand the mechanisms underlying Sarrah’s function, mass spectrometry-based proteomics was employed to identify its interacting protein partners, specifically CRIP2 and p300. This integrative approach demonstrated that Sarrah modulates gene expression by interacting with these proteins, affecting pathways involved in cardiomyocyte survival and endothelial cell function during aging [36]. Additionally, one study employed an RNA-guided proteomics computational pipeline to analyze the mass spectrometry data and detected hundreds of putative splice variant proteins that have the potential to rewire the cardiac proteome [18]. These examples underscore the importance of integrating RNA-seq and proteomics to unveil the real changes at both the transcript and protein levels, leading to a deeper understanding of the molecular mechanisms driving cardiac aging.

For more predictive applications, a recent study integrated plasma protein mapping to bulk RNA seq to estimate organ-specific aging [3]. This approach revealed that heart aging proteins were expressed primarily by cardiomyocytes and established NPPB and TNNT2 along with less well-characterized cardiac proteins, including cardiac myosin light chain (MYL7), peroxidasin-like (PXDNL), and bone morphogenetic protein 10 (BMP10), as clinical markers of acute heart failure. Another study integrated RNA–protein correlation using large proteogenomics datasets and human conservation data to describe aging-related gene signatures [37]. By combining RNA-Seq and proteomics, researchers can gain a comprehensive understanding of the molecular mechanisms underlying cardiac aging, identifying key pathways, proteins, and genetic factors involved in this complex process.

## 6. Conclusions and Prospects

The advent of high-throughput multi-omics techniques has transformed our understanding of heart aging, providing unprecedented insights into the molecular and cellular processes that drive this complex phenomenon. By integrating data from genomics, epigenomics, transcriptomics, proteomics, and metabolomics, researchers can uncover the intricate networks and pathways involved in cardiac aging, enabling the identification of novel biomarkers and therapeutic targets. 

In particular, the use of single-cell and single-nucleus RNA sequencing, alongside chromatin accessibility assays like scATAC-seq, has illuminated the heterogeneity within cardiac cell populations and their distinct aging trajectories. These approaches have revealed critical changes in gene expression and chromatin dynamics, highlighting key regulatory factors and cellular interactions that contribute to the aging process. Moreover, the integration of RNA-seq and proteomics has demonstrated that transcriptomic changes do not always correlate with proteomic alterations, underscoring the need for multi-omics approaches to capture the full scope of molecular changes. Such integrative analyses have identified age-associated pathways and protein complexes, offering deeper insights into the functional consequences of aging at both the transcript and protein levels.

Looking ahead, the continued acquisition and integration of multi-omics datasets will enhance our understanding of cardiac aging. With the increasing number of aging-related datasets, research on integrating information is required to obtain a more robust understanding of their molecular underpinnings and translational potential [51]. One example is the aging clock, which has been used to interpret the complex biology of aging and guide clinical decision making [11] by distilling omics data into composite aging biomarkers to predict aging phenotypes. A tailored cardiac aging clock incorporating multi-omics is likely to help to obtain optimal clock performance and biological relevance [11]. 

Emerging techniques like spatial transcriptomics, though not yet widely applied to aging research, have shown potential in studying cardiac diseases such as myocardial infarction [52,53,54], fibrosis [55], and atherosclerosis [56,57,58]. These techniques could offer new avenues for exploring the spatial organization of aging-related molecular changes within cardiac tissue, further enriching our understanding of the aging heart.

In conclusion, the integration of high-throughput multi-omics approaches represents a powerful strategy for unraveling the complexities of heart aging. As we continue to refine these techniques and expand our datasets, we move closer to translating these insights into effective therapies and interventions, ultimately improving cardiovascular health and longevity.

## Figures and Tables

**Table 1:** Multi-omics studies in cardiac aging.

Omics	Techniques Used	Aging Hallmarks	Samples	Major Findings of Aging Impact	Ref.
Epigenomics	ChIP-seq, Hi-C seq	Epigenetic alteration	murine whole hearts (6, 12, 18 months)	Disruption of nuclear lamina and chromatin architecture leads to the misexpression of genes lacking CpG islands, contributing to chronic inflammation, physiological deterioration, and the loss of functional identity during cardiac aging.	[12]
	ChIP-seq, RNA-seq		male murine left ventricles (6, 24 months)	Changes in chromatin accessibility and histone modifications, active marks (H3K27ac) and repressive marks (H3K27me3), are prominent in the aging cardiomyocytes, with relatively stable transcriptomes but significant epigenetic alterations.	[13]
	ChIP-seq, Hi-C seq, RNA-seq, UHPLC-MS/MS *		murine cardiomyocytes (2, 6, 18 months)	The enhancer activation of glycolysis genes via p300/CBP is a key driver of metabolic remodeling in cardiac aging, and the pharmacological inhibition of p300/CBP can blunt age-related cardiac dysfunction.	[14]
	snATAC-seq		murine hearts, brains, bone marrow, skeletal muscle (3, 10, 18 months)	Cardiac ECs showed uniquely more accessible promoter of Nhp2 during aging, while cardiac cells in general showed fewer age-related changes to accessibility of cCREs than other tissues tested.	[15]
Transcriptomics	Bulk RNA-Seq	Oxidative stress, disrupted metabolism, cellular senescence, chronic inflammation, mitochondrial dysfunction, disabled macroautophagy	zebrafish ventricles (7, 48 months)	Aging hearts showed the upregulation of genes of immune response and chemotaxis, downregulation of genes of metabolism and tissue regeneration, and impaired regenerative capacity to cardiac injury.	[16,17]
	Bulk RNA-Seq MS *		hearts from male and female mice (4, 20 months)	Heart tissues showed sex-specific changes to gene expression with age impacting mitochondrial metabolism, translation, autophagy, etc., and rewiring of RNA splicing programs in exon usage and splice patterns.	[18]
	Bulk RNA-Seq		rat hearts (6, 12, 17, 36 months)	Aging hearts showed upregulation of circadian, senescence and cellular stress genes, and conserved aging-related gene expression patterns across species.	[19]
	Bulk RNA-Seq		zebrafish (2, 7, 16, 39 months); rat (6, 12, 17, 36 months) hearts	Identified conserved and distinctive aging-related changes to gene expression across tissues and species. Cardiac specific changes in fish involved immune response genes, while those in rats involved wound healing and heart development genes.	[20]
	Bulk RNA-Seq		human atrial tissue (adult: 18–65 years and aged: >65 years); rat CMs (6, 24 months)	Increased ROS production in both mitochondrial and extramitochondrial pathways, altered ROS clearance, and reduced antioxidant pathways in both species.	[21]
	Bulk RNA-Seq		rat left ventricular CMs with/without relaxin treatment (9, 24 months)	Aging enhanced inflammation and fibrosis, while relaxin helped to reverse markers. More pronounced aging effects in female hearts can be reduced by relaxin.	[22]
	Bulk RNA-Seq, scRNA-Seq		murine ECs (3, 24 months)	Endothelial Apelin receptor-enriched subtype reduced in aged hearts, downregulation of endothelial barrier function and calcium signaling, upregulation of diabetes and metabolism pathways, and altered receptor-ligand interactions.	[23]
	snRNA-seq		cynomolgus monkey left ventricles (adult: 4–6 years and aged: 18–21 years)	Aged hearts showed upregulated Senescence-Associated Secretory Phenotype (SASP) gene expression, upregulated transcription factors (TFs), FOXP1 and FOXP2, and diminished cytokine production of anti-inflammatory M2 macrophages.	[24]
	snRNA-seq		murine hearts (3, 18 months)	Aged fibroblasts showed increased transcriptional heterogeneity, disrupted interactions with endothelial cells, antiangiogenic properties, and adoption of osteogenic traits.	[25]
	Bulk RNA-Seq, snRNA-Seq		murine hearts (3–7, 18–20 months)	Aging reduces nerve density and dysregulates vascular-derived neuroregulatory genes. Accumulation of senescent cells and downregulation of miR-145 increase semaphorin-3A. Treating aged mice with senolytic drugs could help reverse these effects.	[26]
	scRNA-seq		cynomolgus monkey aortic and coronary arteries (adult: 4–6 years and aged: 18–21 years)	Arterial aging leads to downregulation of FOXO3A, leading to the disruption of vascular homeostasis. Aging also causes the inflammatory response, increased wall thickness, calcification, fibrous cap formation, and fragmentation of elastic lamina.	[27]
	scRNA-seq ATAC-seq		utilized publicly available data for murine and primate aging models	Endothelial cell aging leads to the upregulation of BACH1 in both mouse and monkey vasculature, leading to endothelial dysfunction and senescence; BACH1 binds to open chromatin regions, particularly at the enhancer of the CDKN1A gene, promoting its transcription in senescent endothelial cells.	[28]
Proteomics	UHPLC-MS RNA-seq	Disrupted metabolism loss of proteostasis	male diabetic murine hearts (13 weeks)	LAV-BPIFB4 gene therapy reprograms rather than reverses the diabetic heart phenotype, offering potential cardioprotective effects through subtle metabolic changes.	[29]
Metabolomics	NMR spectroscopy	Dysregulated metabolism	murine hearts, brains, kidneys, livers, lungs and spleens (2–3, 24–26 months)	Aging showed increased GABA in modulating heart rhythm and branched-chain amino acids (BCAAs), such as leucine, isoleucine, valine, and the change in purine metabolites.	[30]
	NMR spectroscopy		female and male murine heart, brain, liver, lung and skeletal muscle tissues (3, 6, 12, 24 months)	Aging process showed biphasic patterns of acetic acid and BCAAs, and sex differences in metabolic alterations.	[31]
			murine hearts and blood with/without mitoxantrone treatment (19 months)	MTX treatment in aged mice led to long-lasting cardiac metabolic adaptations, including increased fatty acid oxidation, reduced glucose metabolism, and amino acid oxidation.	[32]
Multi-Omics	scRNA-seq scATAC seq	All cardiac aging hallmarks	murine aortas (4, 26, 86 weeks)	Aortic aging in mice leads to cell-type specific transcriptional and chromatin accessibility changes. The EC subtype EC1 showed higher involvement in senescence and inflammation, while EC2 was more associated with vascular tone regulation. Key transcription factors, Atf3 and Stat3, were identified as regulators of these processes.	[33]
			male and female human heart left ventricle and/or apex (25–60 years)	Identified sex-and-age-linked molecular signatures, e.g., increased immune activation, metabolic shifts, and pathways changes, such as TGF-β signaling and epithelial-to-mesenchymal transition (EMT), particularly affecting fibroblasts, macrophages, and cardiomyocytes in the human heart.	[34]
	snATAC-Seq snRNA-Seq		male and female mice (6, 12, 18 months)	Aging in the mouse heart is associated with changes in immune response, protein homeostasis, and cellular transport, with significant impacts on protein folding, fatty acid oxidation, and autophagy.	[35]
	RNA-seq LC/MS *		murine hearts (2–3 months, 18 months); human engineered heart tissue	The lncRNA Sarrah is a conserved anti-apoptotic regulator that is downregulated during cardiac aging and promotes cardiomyocyte survival after ischemic injury.	[36]
	RNA-seq LC/MS		male and female murine cardiac and skeletal muscle (4, 20 months)	Cardiac aging showed a significant shift in gene expression from mitochondrial metabolism and protein synthesis towards immune activation and extracellular matrix remodeling. About 48% of the aging-associated transcriptomic changes in the heart is predictive of protein-level changes.	[37]

* UHPLC–MS/MS: ultra–performance liquid chromatography–mass spectrometry; MS: mass spectrometry; LC/MS: liquid chromatography/mass spectrometry.
